# A Unique Presentation of a Large, Seven-segment, Spinal Epidural Abscess in a Patient with a Pleural Empyema

**DOI:** 10.7759/cureus.4084

**Published:** 2019-02-16

**Authors:** Kyle L Barner, Kyle M Yuquimpo, David A McMillan, Evan M Shaw

**Affiliations:** 1 Internal Medicine, Kansas City University of Medicine and Biosciences, Kansas City, USA; 2 Internal Medicine, University of Kansas Medical Center, Kansas City, USA

**Keywords:** spinal epidural abscess, epidural abscess, motor weakness, paresthesias, spinal canal, infection, pyogenic infection, spinal cord compression

## Abstract

Spinal epidural abscesses are insidious infections spread via hematologic, contiguous, or iatrogenic routes. On average, spinal epidural abscesses span two to four vertebral segments and are most commonly localized to the thoracic region. Fever, back pain, and neurological deficits are the most common clinical manifestations. However, the triad of these findings are not always detected. Patients may present with subtle symptoms leading to misdiagnosis and poor prognosis. We present a case of a large, anteriorly located, spinal epidural abscess in a patient originally admitted for dyspnea and confusion.

## Introduction

Spinal epidural abscesses (SEA) are generally rare in prevalence. A single-center study by Vakili and Crum-Ciaflone reported SEA in five of every 10,000 admissions, with the highest prevalence occurring in patients between the ages of 50 and 70 years [1]. The incidence of SEA appears to be rising due to an increase in the sensitivity and specificity of current imaging modalities (e.g. MRI) [2-3]. Hematologic spread accounts for a large proportion of SEA cases, and they are usually bacterial in etiology; the most commonly cultured species being Staphylococcus aureus [3]. Diabetes mellitus, intravenous (IV) drug use, and local/systemic infections (e.g., respiratory, urinary, soft-tissue) are other important sources for SEA. Interestingly, a significant portion of the cases were found not to have an identifiable cause for infection [3]. A meta-analysis of thoracic SEA cases by Howie et al. reported the most common symptoms to be neurological deficits (68%), back pain (64%), and fever (24%); however, the triad of these findings are nonspecific for SEA [4]. MRI is considered the gold standard for diagnosis, with most of the abscesses being detected ranging from two to four spinal segments in size [3,5]. We present a unique case of a SEA two to three times larger than average found in a middle-aged male who initially presented to the emergency department (ED) for dyspnea and confusion.

## Case presentation

**Figure 1:**
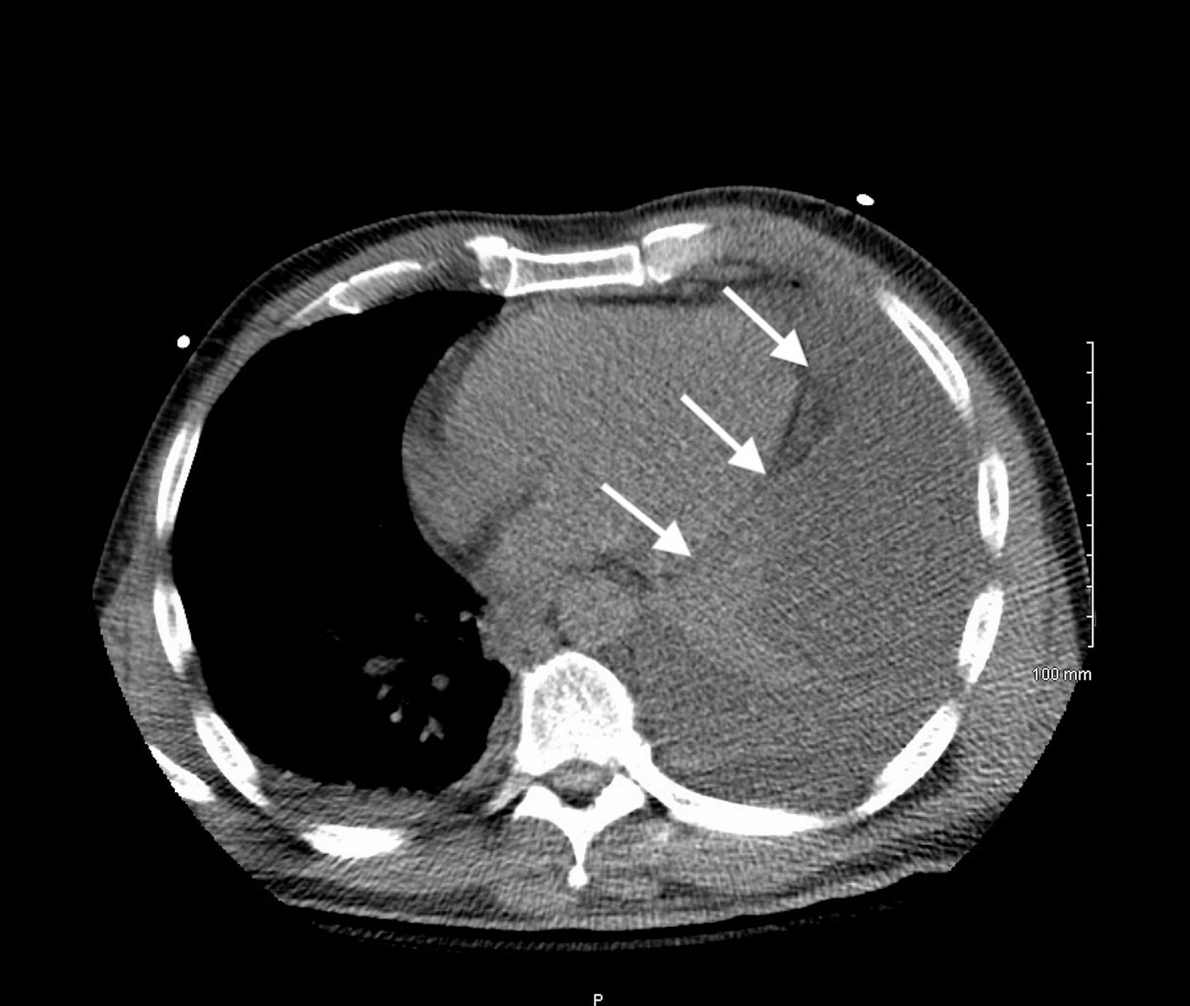
Axial image from non-contrast CT of the chest showing a large, loculated, left-sided pleural effusion bordering the posterolateral margin of the heart (arrows). CT = computed tomography

A 55-year-old male with a 40 pack-year smoking history, hepatitis C, and extensive IV drug use presented to the emergency department (ED) complaining of hip pain and mild shortness of breath after falling on his side in his home. The patient was admitted and an initial computed tomography (CT) scan revealed a small, left-sided pleural effusion. After appearing stable on medical observation, the patient was discharged after one day with pain medication for his symptoms. Three days later, the patient again presented to the ED with worsening dyspnea, confusion, and continuing left-sided hip pain. Physical examination was positive for confusion and unequal pupils. The patient denied any subjective fevers, but stated that he had experienced sweats prior to admission. Objectively, the patient’s vital signs showed an oxygen saturation of 86% on room air, though his respiratory rate and temperature were within normal limits at the time. A large, loculated, left-sided pleural effusion was revealed on non-contrast CT of the chest (Figure 1).

Initial labs revealed no leukocytosis, but they did reveal a mildly elevated serum lactic acid of 2.3 mmol/L (normal: <2.0 mmol/L). Additionally, urine screen was positive for amphetamines, benzodiazepines, and opiates. Later that day, the patient became febrile (39.0 Celsius) and tachypneic (40-50 breaths per minute). He was subsequently transferred to the medical intensive care unit for acute hypoxic respiratory failure and placed on 10-15 liters of high-flow oxygen and empirically treated with levofloxacin and piperacillin/tazobactam. The day after admission, ultrasound-guided thoracentesis was performed revealing bloody fluid containing 20,000 white blood cells (WBC)/microliter (normal: <1,000 cells/microliter) with 95% neutrophils, a pH of 6.91 (normal: 7.60-7.64), and lactate dehydrogenase (LDH) of 7,827 U/L (serum LDH: 559 U/L). Additionally, the patient's serum WBC count was now elevated at 15,000 cells/microliter and blood cultures were positive for methicillin-sensitive Staphylococcus aureus (MSSA), prompting a switch in antibiotics to vancomycin and ampicillin/sulbactam. Four days after admission, the patient underwent bronchoscopy and video-assisted thoracic surgery (VATS) for decortication of the suspected loculated empyema. Status-post VATS, the patient's fever and leukocytosis began to resolve. Empiric antibiotics were discontinued and the patient was started on intravenous (IV) cefazolin.

**Figure 2:**
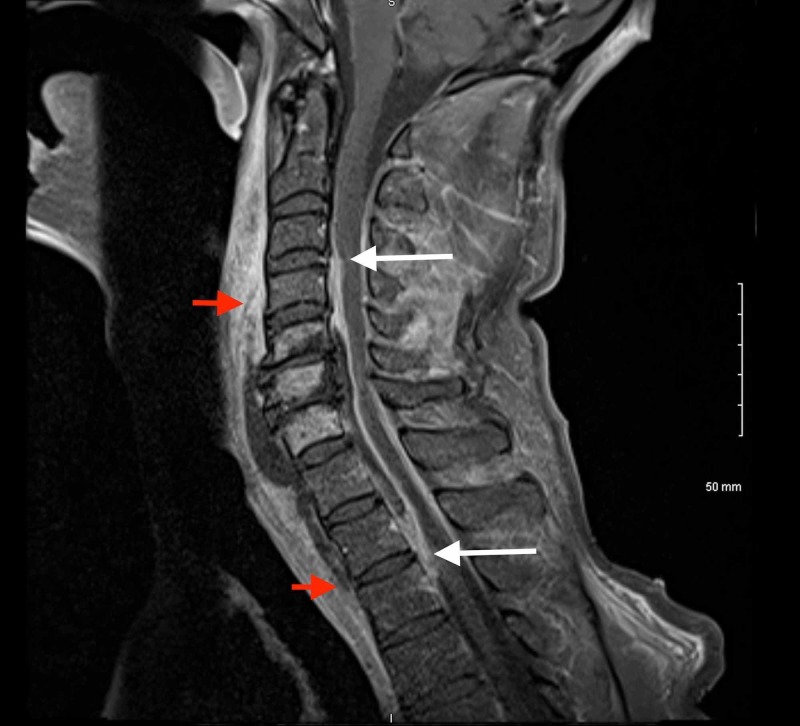
Sagittal T1 post-contrast image of the cervicothoracic spine showing a fluid collection in the anterior epidural space from C3 to T2 (white arrows), and prevertebral fluid collection from C4 to T2 (red arrows). MRI = magnetic resonance imaging

Four days status-post VATS (eight days status-post admission), the patient began to exhibit subtle right-sided shoulder pain and numbness extending into the right hand. Initial non-contrast CT scan revealed posterior C6-C7 osteophytic spurring, disc bulging, and moderate spinal stenosis. A cervicothoracic magnetic resonance imaging (MRI) scan with contrast was attempted but was not completed due to the patient being agitated and anxious during the imaging process. Eight days status-post VATS, the patient reported increasing weakness and 'cramp-like sensations' in all extremities at rest. Additionally, the patient complained of plantar numbness in the feet, decreased sensation to light touch in bilateral upper extremities, and neck pain radiating to the thoracic and lumbar spine. Neurology was consulted and a focal neurological exam found decreased grip strength and clonus of the knees and ankles. The patient was prescribed gabapentin for symptomatic treatment. Cervical and thoracic MRI with contrast were performed, which revealed fluid collections with circumferential dural thickening and enhancement spanning spinal segments C3 to T2 in the anterior epidural space, prevertebral fluid collection spanning from C4 to T2, retropharyngeal fluid and edema from C1 to T3, discitis and osteitis from C5 to C7, and spinal cord narrowing from C4 to C6 with signal abnormality consistent with compressive myelitis (Figure 2). These findings were concerning for SEA given the patient's positive blood cultures for MSSA, IV drug use, and previously diagnosed empyema.

A decompression laminectomy and washout of purulent fluid and thick phlegmon in the epidural space from C1 to T3 was performed by neurosurgery the day after diagnostic MRI. Culture and sensitivity testing of the purulent fluid from the spinal epidural space revealed MSSA.

The day after spinal decompression and laminectomy, the patient's WBC count dropped back into the normal range and remained there for the rest of the hospitalization. However, the patient reported continued extremity weakness and paresthesia that only partially improved with gabapentin. The patient was discharged for physical rehabilitation. Over the next few days, the patient’s arm strength improved and he required minimal assistance with daily activities. The patient continued to improve but had persistent upper extremity pain and weakness and was followed by the internal medicine and infectious disease services throughout the course of his antibiotic treatment with IV cefazolin.

## Discussion

Spinal epidural abscesses are a generally rare and insidious condition leading to difficulty in diagnosis, with up to 17% of patients being initially misdiagnosed most commonly for meningitis or intervertebral disc prolapse [2,6]. The patient initially exhibited signs of neural involvement on day nine of admission with mild right shoulder pain and numbness extending into his right hand. The three most common indications of SEA are back pain, fever, and neurological deficit by mechanism of direct mechanical compression or indirect spinal cord ischemia secondary to thrombophlebitis [7]. However, heavy dependence on clinical signs or symptoms is not reliable as only a minority of patients with SEA present with these symptoms [8]. Status-post VATS procedure for empyema, the patient’s elevated WBC count began to decrease, and he eventually became afebrile, thus not warranting an emergent MRI. The patient underwent MRI studies at the onset of his right-hand weakness, but due to poor patient compliance, these studies were not successfully completed until four days after the initial neurological presentation. Diagnosis of the cervicothoracic SEA was made via MRI after a previous cervical CT was performed not showing evidence of SEA. The utilization of CT in the diagnosis of SEA is known to be limited due to poor sensitivity and limited differentiation between the borders of the spinal cord and epidural space [2].

The most important prognostic factor in SEA is the time course to diagnosis and treatment. Prolonged compression of the spinal cord may lead to irreversible damage including paralysis and death [9]. Urgent surgical spinal decompressive laminectomy and washout is paramount and should be performed within 24-36 hours of the onset of paralytic symptoms to reduce the risk of suffering irreversible sequelae [7]. Surgical intervention was appropriate for this case, as the patient was experiencing rapid and extensive neurological decline and symptoms for at least 96 hours. The patient continued to have weakness and paresthesia in all four extremities, likely due to irreversible ischemia secondary to mechanical compression by the SEA. 

The triggering factors for the sudden presentation of the patient’s symptoms are unknown; however, comorbidities such as pulmonary and mediastinal infections, sepsis, and IV drug use have been associated with an increased risk of developing SEA. A significant proportion of patients developing SEA were also found to have previously undergone invasive procedures such as spinal epidural anesthesia [2]. Despite having almost all of the previously mentioned associative factors, as well as having an SEA two to three times the average size, the patient initially only exhibited subtle neurological symptoms. Imaging of the patient revealed a SEA located in the anterior region of the spine. The majority of anteriorly-located SEA are caused by direct anatomical spread and are associated with concomitant osteomyelitis, while posteriorly located SEA are frequently caused by hematologic seeding [8,10]. Furthermore, the SEA discovered was located in the cervicothoracic region of the spine. The epidural space is limited down to C7 due to the thickening of the spinal cord in the cervical region compared with the thoracic and lumbar regions which have a larger epidural space as well as an extradural venous plexus that acts as a potential conduit for bacterial spread. This anatomy lends to the majority of SEA localizing outside of the cervical region [2,11]. A study by Reihsaus et al., observing 915 cases of SEA, showed that SEA only localized to the cervical and cervicothoracic region 19% and 7% of the time, respectively [2].

## Conclusions

Patients presenting with subtle changes in neurological status, fever of unknown etiology, back pain, risk factors such as bacteremia, IV drug use, history of recent respiratory infection, or an invasive procedure should prompt immediate investigation into the possibility of spinal epidural abscess development. Quick action and surgical management early in the disease process is critical for halting and possibly reversing neurological decline and can drastically change the patient’s prognosis.
